# Hemodynamic implications of mitral annular calcification in patients undergoing transcatheter aortic valve implantation for severe aortic stenosis

**DOI:** 10.1007/s10554-023-02931-w

**Published:** 2023-10-06

**Authors:** Kensuke Hirasawa, Steele C. Butcher, Ana Rita Pereira, Maria Chiara Meucci, Jan Stassen, Philippe van Rosendael, Nina Ajmone Marsan, Jeroen J Bax, Victoria Delgado

**Affiliations:** 1 Department of Cardiology, Leiden University Medical Center, Leiden, 2300RC The Netherlands; 2 Department of Cardiovascular Medicine, Tokyo Medical and Dental University, Tokyo, Japan; 3 Department of Cardiology, Royal Perth Hospital, Perth, WA Australia; 4 Department of Cardiology, Hospital Garcia de Orta, Almada, Portugal; 5 Department of Cardiovascular and Thoracic Sciences, Fondazione Policlinico Universitario A. Gemelli IRCCS, Catholic University of the Sacred Heart, Rome, Italy; 6 Heart Center, University of Turku, Turku University Hospital, Turku, Finland; 7 Department of Cardiology, Heart Lung Center, Albinusdreef 2, Leiden, 2300 RC the Netherlands

**Keywords:** Transcatheter aortic valve intervention, Aortic stenosis, Mitral annular calcification, Mitral stenosis, Hemodynamics

## Abstract

**Purposes:**

Predicting hemodynamic changes of stenotic mitral valve (MV) lesions with mitral annular calcification (MAC) following transcatheter aortic valve implantation (TAVI) may inform clinical decision-making. This study aimed to investigate the association between the MAC severity quantified by computed tomography (CT) and changes in mean transmitral gradient (mTMG), mitral valve area (MVA) and stroke volume index (SVi) following TAVI.

**Methods and results:**

A total of 708 patients (median age 81, 52% male) with severe aortic stenosis (AS) underwent pre-procedural CT and pre- and post-TAVI transthoracic echocardiography. According to the classification of MAC severity determined by CT, 299 (42.2%) patients had no MAC, 229 (32.3%) mild MAC, 102 (14.4%) moderate MAC, and 78 (11.0%) severe MAC. After adjusting for age and sex, there was no significant change in mTMG following TAVI (Δ mTMG = 0.07 mmHg, 95% CI -0.10 to 0.23, P = 0.92) for patients with no MAC. In contrast, patients with mild MAC (Δ mTMG = 0.21 mmHg, 95% CI 0.01 to 0.40, P = 0.018), moderate MAC (Δ mTMG = 0.31 mmHg, 95% CI 0.02 to 0.60, P = 0.019) and severe MAC (Δ mTMG = 0.43 mmHg, 95% CI 0.10 to 0.76, P = 0.0012) had significant increases in mTMG following TAVI, with greater changes associated with increasing MAC severity. In contrast, there was no significant change in MVA or SVi following TAVI.

**Conclusion:**

In patients with severe AS undergoing TAVI, MAC severity was associated with greater increases in post-procedural mTMG whereas MVA or SVi remained unchanged. MAC severity should be considered for potential subsequent MV interventions if TAVI does not improve symptoms.

**Supplementary Information:**

The online version contains supplementary material available at 10.1007/s10554-023-02931-w.

## Introduction

Multiple valve disease is frequently observed in patients undergoing transcatheter aortic valve implantation (TAVI), with significant mitral stenosis (defined as a mean transmitral pressure gradient [TMG] ≥ 5 mm Hg) discernible in approximately 10% of patients [1, 2]. Predicting alterations to the hemodynamic significance of concomitant mitral valve lesions following TAVI may inform clinical decision-making and provide an insight into underlying valvular pathophysiology. Indeed, changes in mitral regurgitation following TAVI for severe aortic stenosis (AS) have been well-described, with studies describing a reduction in regurgitation severity in 50–70% of patients [3, 4]. However, alterations in valvular hemodynamics following TAVI that are associated with mitral stenosis remain poorly understood [5]. For instance, it is unclear whether the mean TMG increases or decreases following the procedure, or whether there will be a reduction or improvement in the hemodynamic impact of mitral valve stenosis [5–7]. Furthermore, studies which have examined changes in these hemodynamic indices have compared pre- with early post-procedural echocardiography, a time susceptible to temporary hemodynamic derangements due to procedural complications and the use of inotropes, with delays in the recovery of normal loading conditions [6, 8–10].

A method of quantifying mitral annular calcification (MAC) utilizing cardiac computed tomography (CT) has previously been demonstrated to identify patients at risk of poor outcome during follow-up after TAVI [11]. However, whether the severity of MAC is associated with alterations in mitral valve hemodynamics following TAVI remains unclear. Therefore, this study aimed to investigate the association between the severity of MAC quantified by cardiac CT and changes in mean TMG, mitral valve area (MVA) and stroke volume index (SVi) in the short- to medium- term following TAVI.

## Materials and methods

### Study population

Patients with severe AS who underwent TAVI at the Leiden University Medical Centre between November 2007 and December 2019 were selected from the departmental echocardiographic database. Those who underwent valve-in-valve TAVI or had previous mitral valve surgery/intervention were excluded. In addition, patients without available pre-TAVI cardiac CT images, pre- and/or post-procedural echocardiographic images and who had pre- and/or post-procedural tachycardia (defined as a heart rate > 100 bpm) were excluded. Patient demographic and clinical data were retrieved from the departmental electronic medical record (EPD-vision; Leiden University Medical Centre, Leiden, the Netherlands).

### Cardiac computed tomography data acquisition and mitral annulus calcification assessment

**Fig. 1:**
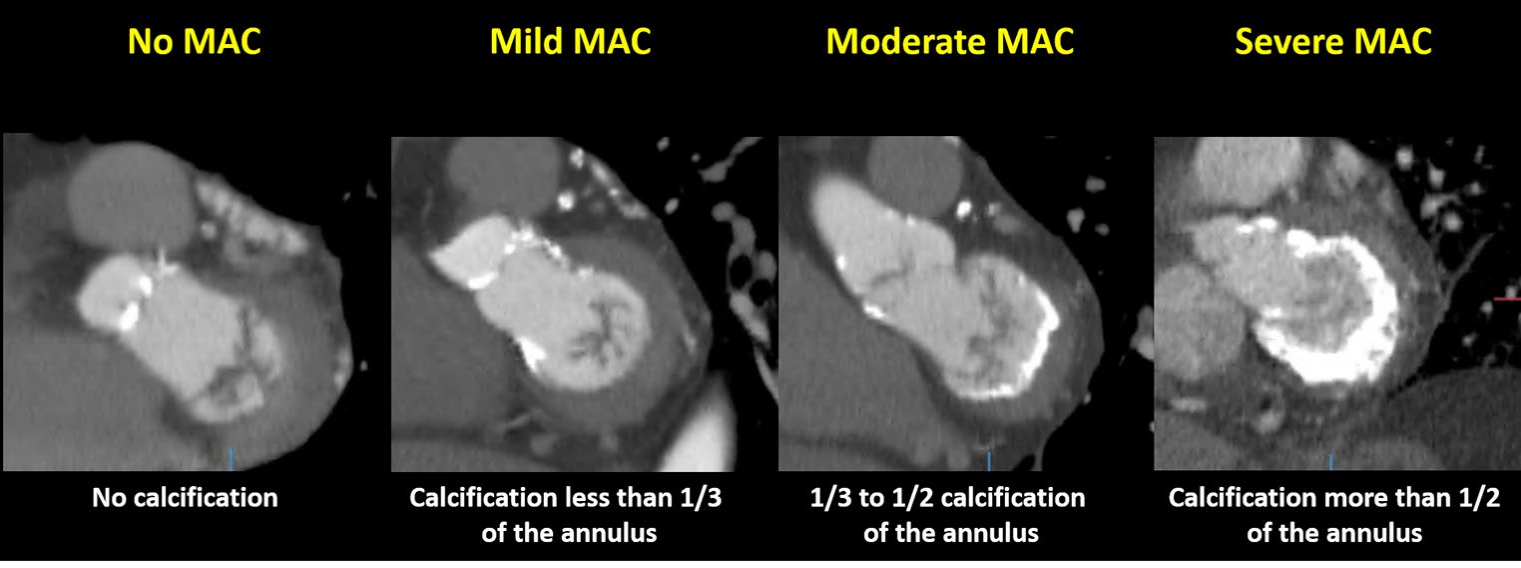
**CT Evaluation of MAC**. MAC severity was quantitively determined according to the circumferential involvement of calcification in mitral annulus MAC = mitral annular calcification

Cardiac CT data were acquired prior to TAVI using a 64-row (Aquilion64, Toshiba Medical Systems, Otawara, Japan) or a 320-row CT scanner (AquilionOne, Toshiba Medical Systems, Tochigi-ken, Japan). With a 64-detector scanner, data acquisition was performed gated to the ECG to facilitate retrospective gating. In contrast, with a 320-detector scanner, the entire cardiac cycle was scanned using prospective ECG triggered dose modulation. Data processing was performed using offline CT workstations (3mensio version 10.2, Pie Medical Imaging, Bilthoven, the Netherlands; Vitrea 2, Vital Images, Plymouth, MN, USA). MAC was qualitatively and quantitatively assessed by 2 observers (K.H and A.R.P). MAC severity was determined visually according to the degree of circumferential involvement of the mitral annulus on axial slices, as follows: no MAC = no calcification of the mitral annulus; mild MAC = calcification less than 1/3 of the mitral annulus; moderate MAC = calcification between 1/3 and 1/2 of the mitral annulus; and severe MAC = calcification of more than 1/2 of the mitral annulus (Fig. 1) [11]. Quantitative assessment of MAC was performed using dedicated offline workstation (Vitrea 2, Vital Images, Plymouth, MN, USA). The software identified calcification through the detection of pixels ≥ 130 Hounsfield Units (HU). The degree of MAC was quantified automatically within region of interest as the Agatston score, as previously described [12, 13].

### Echocardiography

Comprehensive transthoracic echocardiography was performed using a Vivid 7, E9, or E95 ultrasound systems (General Electric Vingmed Ultrasound, Horten, Norway) with patients at rest in the left lateral decubitus position. Electrocardiogram-triggered echocardiographic data were acquired with 3.5 MHz and M5S transducers and stored digitally in a cine-loop format for offline analysis with dedicated software (EchoPAC Version 203, 204, General Electric Vingmed Ultrasound, Horten, Norway). Echocardiographic images used for the evaluation of mitral valve hemodynamics at follow-up were acquired at approximately 1 and 6 months after TAVI, as per the institutional protocol. AS severity was evaluated according to peak aortic jet velocity, mean pressure gradient and aortic valve area [14]. Left ventricular (LV) outflow tract diameter was measured using a zoomed parasternal long-axis view, immediately proximal to the aortic valve. The LV outflow tract velocity-time integral was measured on pulsed-wave Doppler recordings from an apical three- or five-chamber view, and was used to calculate stroke volume, which was indexed to body surface area. Aortic and mitral regurgitation severity were graded according to contemporary guideline recommendations as none, mild, moderate or severe, using a multiparametric integrative approach [15]. Mean TMG and the mitral velocity-time integral were derived from transmitral flow recorded with continuous wave Doppler. MVA was calculated using the continuity equation, dividing stroke volume by the mitral velocity-time integral. LV ejection fraction was calculated using the biplane Simpson method, while LV mass was quantified using a 2-dimensional linear approach [16]. Left atrial volume was measured on apical two- and four-chamber views using the Simpson method and was indexed for body surface area. All other standard measurements were performed according to the American Society of Echocardiography and European Association of Cardiovascular Imaging guidelines [16].

### Statistical analysis

Categorical variables are presented as numbers and percentages, while continuous variables are presented as median and interquartile range (IQR). Differences between the four groups defined by MAC severity were analyzed using the Pearson χ^2^ test for categorical variables and the Kruskal-Wallis test for continuous variables. Multiple comparisons were tested using Bonferroni’s correction.

Linear mixed models with a random per patient intercept were used to determine the association between MAC and changes in mean TMG, MVA and SVi over time, while adjusting for age and sex. The interactions between MAC severity and time (pre- and post- TAVI echocardiography) were reported for each model. Estimated marginal means with 95% confidence intervals (CIs) for mean TMG, MVA and SVi were estimated using the Kenward-Roger method for each mixed model, according to MAC group and at pre- and post- TAVI time points. P-values comparing pre- and post- TAVI estimated marginal means for each hemodynamic parameter according to MAC group were estimated using *t*-tests for pairwise comparisons, adjusted for multiple comparisons using the Tukey method. In addition, spline curves were fitted to investigate the association between Agatston score (quantified with cardiac CT) of MAC and pre-TAVI and post-TAVI mean TMG. A linear mixed model with a random per patient intercept was used to evaluate the association between MAC quantified by the Agatston score and changes in mean TMG, adjusting for age and sex.

All tests were two-sided, with *P*-values < 0.05 considered statistically significant. Statistical analysis was performed using SPSS version 25.0 (IBM Corporation, Armonk, NY, USA) and R version 4.0.1 (R Foundation for Statistical Computing, Vienna, Austria).

## Results

### Clinical and echocardiographic characteristics

**Fig. 2:**
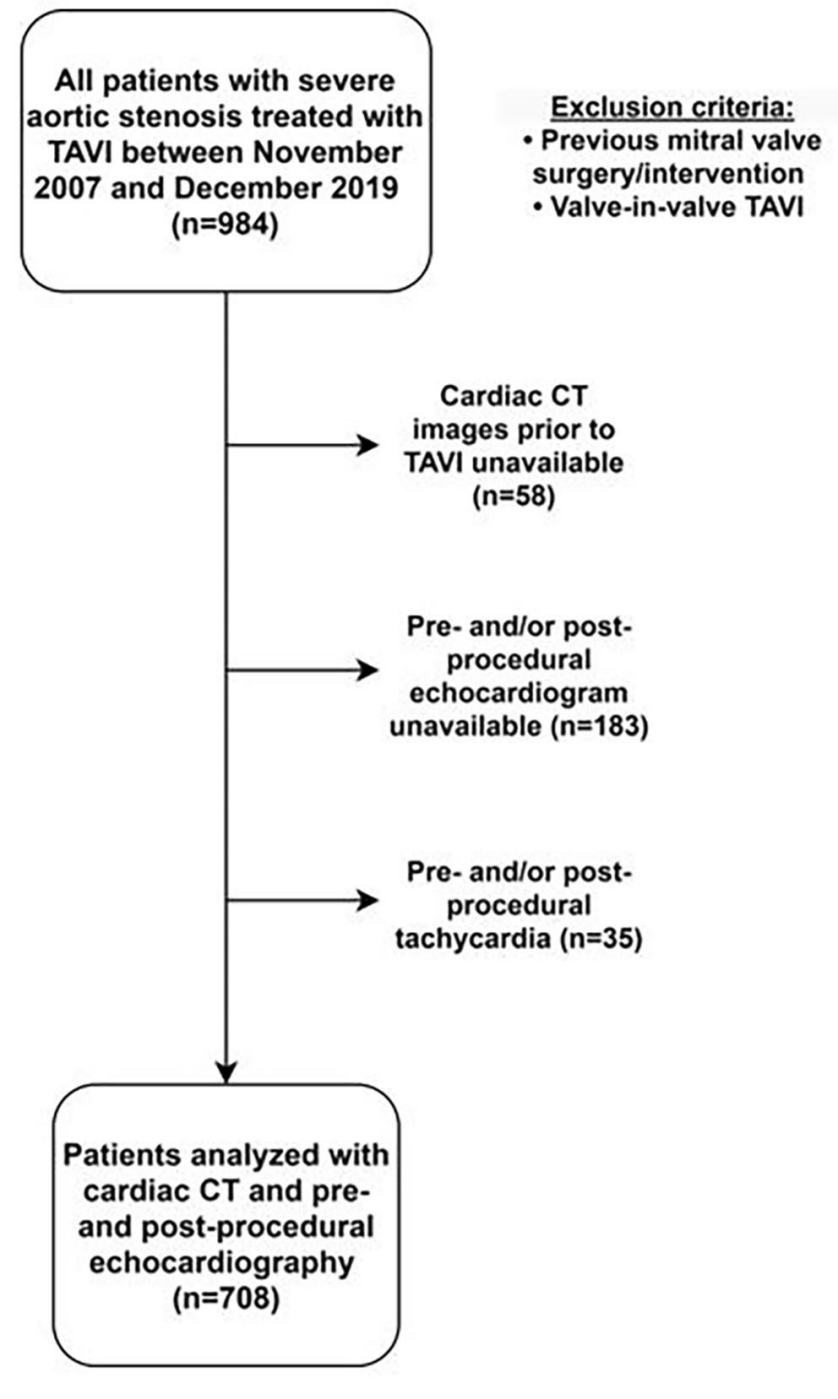
**Study Flow Chart** CT = computed tomography; TAVI = transcatheter aortic valve implantation

A total of 708 patients with severe AS who successfully underwent TAVI were included (Fig. 2). The median age of the population was 81 (IQR, 76 to 85) years and 52% were male. A total of 388 (55%) patients reported New York Heart Association class III or IV heart failure symptoms. According to the classification of MAC severity by cardiac CT, 299 (42.2%) patients had no MAC, 229 (32.3%) mild MAC, 102 (14.4%) moderate MAC, and 78 (11.0%) severe MAC. Quantification of Agatston score for MAC with cardiac CT was feasible in 540 (76%) patients. The median Agatston score for MAC was 0 (IQR, 0 to 0) for patients with no MAC, 691 (IQR, 379 to 1357) for those with mild MAC, 3024 (IQR, 1913 to 3928) for patients with moderate MAC and 8296 (IQR, 4973 to 12,968) for those with severe MAC. Patients with severe MAC were more likely to be female and had a higher prevalence of previous stroke/transient ischemic attack. The clinical and demographic characteristics of the population are presented in Table 1.

**Table 1:** Patient Characteristics

Variable	Overall, N = 708	No MAC, N = 299	Mild MAC, N = 229	Moderate MAC, N = 102	Severe MAC, N = 78	*P*-value
Age, years	81 (76–85)	81 (76–84)	81 (76–85)	82 (77–85)	83 (78–86)	0.25
Male sex, %	371 (52%)	172 (58%)	131 (57%)	49 (48%)	19 (24%)^*†§^	< 0.001
BMI (kg/m^2^)	25.9 (23.7–28.7)	25.7 (23.7–28.6)	26.0 (24.1–29.0)	26.0 (23.4–28.9)	25.7 (23.5–28.6)	0.69
Systolic blood pressure (mmHg)	138 (121–150)	136 (119–150)	139 (122–150)	140 (124–151)	139 (125–154)	0.22
Diastolic blood pressure (mmHg)	67 (60–75)	66 (60–75)	65 (59–76)	67 (60–74)	69 (61–75)	0.67
Hypertension, %	527 (75%)	222 (74%)	176 (77%)	72 (71%)	57 (75%)	0.69
Dyslipidemia, %	451 (64%)	192 (64%)	145 (63%)	66 (65%)	48 (62%)	0.98
Diabetes mellitus, %	196 (28%)	78 (26%)	59 (26%)	37 (36%)	22 (28%)	0.21
Previous stroke/TIA, %	115 (19%)	52 (21%)	27 (14%)	17 (20%)	19 (30%)^§^	0.049
Coronary artery disease, %	424 (60%)	187 (63%)	134 (59%)	62 (61%)	41 (53%)	0.42
Atrial fibrillation, %	161 (23%)	62 (21%)	64 (28%)	15 (15%)	20 (26%)	0.039
COPD, %	140 (21%)	53 (19%)	50 (23%)	23 (24%)	14 (18%)	0.57
NYHA class III or IV, %	388 (55%)	158 (53%)	131 (58%)	57 (56%)	42 (54%)	0.77
Beta-blocker, %	420 (59%)	182 (61%)	144 (63%)	54 (53%)	40 (51%)	0.15
ACEi or ARB, %	385 (54%)	156 (52%)	135 (59%)	54 (53%)	40 (51%)	0.41
Diuretics, %	388 (55%)	152 (51%)	134 (59%)	57 (56%)	45 (58%)	0.32
Statin, %	458 (65%)	193 (65%)	145 (63%)	73 (72%)	47 (60%)	0.40
P2Y12 inhibitor, %	237 (33%)	101 (34%)	78 (34%)	35 (34%)	23 (29%)	0.89
Oral anticoagulation, %	250 (36%)	98 (33%)	86 (38%)	35 (34%)	31 (41%)	0.53
eGFR (mL/min/1.73m^2^)	66 (50–83)	67 (51–82)	68 (51–86)	63 (48–75)	62 (48–77)	0.18
Hemoglobin (mmol/L)	7.80 (7.10–8.50)	7.90 (7.20–8.60)	7.85 (7.00–8.50)	7.85 (6.90–8.40)	7.55 (6.82–8.12)*	0.005

Median (IQR); n (%)

*p < 0.05 vs. Group I;†p < 0.05 vs. Group II;§p < 0.05 vs. Group III

ACEi = Angiotensin converting enzyme inhibitor; ARB = angiotensin receptor blocker; BMI = body mass index; COPD = chronic obstructive pulmonary disease; eGFR = estimated glomerular filtration rate; MAC = mitral annular calcification; NYHA = New York Heart Association; TIA = transient ischemic attack

Transthoracic echocardiography was performed at a median of 1 (IQR, 1–38) day before TAVI and 35 (IQR, 31–45) days after TAVI. The baseline echocardiographic characteristics of the study population are summarized in Table 2. Patients with severe MAC had smaller LV dimensions and larger LA dimensions compared to those with less severe MAC. However, LV mass, aortic mean pressure gradient and aortic valve area were similar across groups. Increasing severity of MAC was associated with higher values of mean TMG and lower values of MVA. There was no significant difference in the prevalence of moderate or severe mitral regurgitation between groups.

**Table 2:** Echocardiographic characteristics at baseline

Variable	Overall, N = 708	No MAC, N = 299	Mild MAC, N = 229	Moderate MAC, N = 102	Severe MAC, N = 78	*P*-value
Heart rate, bpm	69 (61–77)	67 (60–76)	68 (61–74)	71 (63–78)	70 (63–78)	0.044
LV end-diastolic diameter index, mm/m^2^	24.9 (22.3–28.0)	24.6 (22.5–28.1)	25.0 (21.8–27.5)	25.4 (22.1–28.5)	24.8 (23.0–26.8)	0.65
LV end-systolic diameter index, mm/m^2^	17.4 (14.1–20.7)	17.6 (14.4–21.0)	17.6 (14.5–20.9)	17.1 (14.1–20.7)	15.5 (12.6–18.5)^*†^	0.008
LV mass index, g/m^2^	121 (99–145)	117 (96–143)	124 (102–149)	126 (103–146)	120 (101–145)	0.31
LV end diastolic volume index, ml/m^2^	48 (38–61)	50 (39–67)	50 (40–62)	48 (37–61)	38 (30–46)^*†§^	< 0.001
LV end systolic volume index, mm/m^2^	19 (13–31)	20 (12–32)	22 (14–34)	19 (14–28)	15 (11–21)^*†§^	< 0.001
LV ejection fraction, %	58 (48–65)	59 (48–66)	57 (46–64)	58 (51–66)	61 (53–67)^†^	0.025
Left atrial volume index, ml/m^2^	40 (30–51)	36 (27–46)	41 (31–52)^*^	42 (32–52)^*^	48 (40–63)^*†§^	< 0.001
Stroke volume index, ml/m^2^	38 (31–47)	39 (32–48)	38 (31–47)	38 (32–45)	39 (31–48)	0.78
Aortic peak velocity, m/s	3.97 (3.44–4.48)	3.96 (3.43–4.41)	3.95 (3.40–4.47)	4.00 (3.47–4.38)	4.15 (3.66–4.72)	0.16
Aortic mean pressure gradient, mmHg	41 (31–52)	40 (31–51)	40 (30–51)	40 (29–51)	44 (32–56)	0.29
Aortic valve area, cm	0.78 (0.63–0.94)	0.78 (0.64–0.92)	0.80 (0.63–0.95)	0.77 (0.61–0.96)	0.75 (0.62–0.92)	0.82
Moderate or severe aortic regurgitation	120 (17%)	38 (13%)	46 (20%)	23 (23%)	13 (17%)	0.049
Mean mitral pressure gradient, mmHg	2.08 (1.47–3.05)	1.64 (1.23–2.29)	2.10 (1.49–2.88)^*^	2.95 (1.99–3.84)^*†^	3.55 (2.53–5.24)^*†^	< 0.001
Mitral valve area, cm	2.38 (1.88–2.99)	2.71 (2.09–3.21)	2.38 (1.94–2.88)^*^	2.16 (1.73–2.68)^*†^	1.81 (1.48–2.28)^*†§^	< 0.001
Moderate or severe mitral regurgitation	141 (20%)	57 (19%)	49 (21%)	17 (17%)	18 (23%)	0.67
Mitral inflow E wave velocity, cm/s	85 (64–109)	71 (55–92)	88 (69–109)^*^	95 (70–118)^*^	121 (97–141)^*†§^	< 0.001
Mitral inflow E/A ratio	0.77 (0.60–1.13)	0.72 (0.58–1.04)	0.80 (0.60–1.16)	0.72 (0.58–0.99)	0.93 (0.71–1.22)^*§^	0.001
Tricuspid regurgitation maximal velocity, m/s	2.62 (2.32–3.00)	2.60 (2.29–3.00)	2.67 (2.39–3.02)	2.63 (2.29–2.92)	2.62 (2.38–2.98)	0.48

Median (IQR); n (%)

*p < 0.05 vs. Group I;†p < 0.05 vs. Group II;§p < 0.05 vs. Group III

LV = left ventricle; MAC = mitral annular calcification

### Association between MAC severity and changes in mitral valve hemodynamics following TAVI

**Fig. 3:**
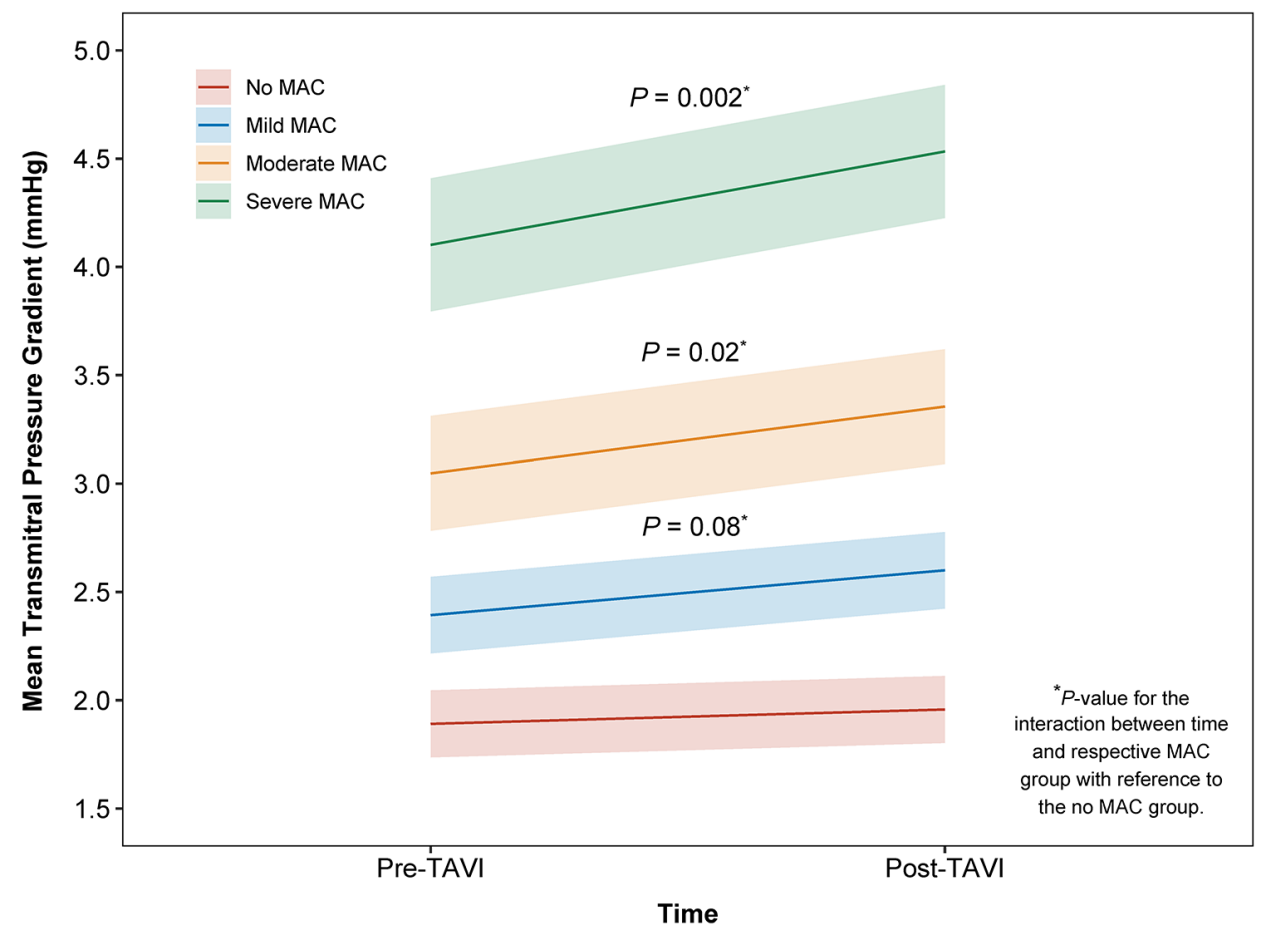
**Changes in mean transmitral pressure gradient following TAVI according to MAC severity**. The predicted means for mean TMG and respective 95% confidence intervals (lightly shaded regions) are displayed according to MAC group. The differences in slopes between MAC groups reflect the interaction between MAC group and time. The *P*-values for the interaction between time and MAC group with reference to the no MAC group are displayed above the corresponding group MAC = mitral annular calcification; TAVI = transcatheter aortic valve implantation; TMG = transmitral pressure gradient

In a model adjusted for age and sex, there was no significant change in mean TMG following TAVI (Δ mean TMG = 0.07 mmHg, 95% CI -0.10 to 0.23, *P* = 0.92) for patients with no MAC. In contrast, patients with mild MAC (Δ mean TMG = 0.21 mmHg, 95% CI 0.01 to 0.40, *P* = 0.018), moderate MAC (Δ mean TMG = 0.31 mmHg, 95% CI 0.02 to 0.60, *P* = 0.019) and severe MAC (Δ mean TMG = 0.43 mmHg, 95% CI 0.10 to 0.76, *P* = 0.0012) had significant increases in mean TMG following TAVI, with greater changes associated with increasing severity of MAC (Table 3). For values of mean TMG, there was a significant interaction between time and the severe (P = 0.002) and moderate MAC groups (P = 0.02), but not the mild MAC group (P = 0.08) with reference to the no MAC group (Fig. 3). Spline curve analysis demonstrated that increasing Agatston score of MAC quantified with cardiac CT was associated with higher values of pre- and post- TAVI mean TMG (Figure S1). In addition, for values of mean TMG, there was a significant interaction between time and Agatston score of MAC (P = 0.017) in a model adjusted for age and sex.

**Table 3:** Estimated marginal means of mean transmitral pressure gradient according to MAC severity

Severity of MAC	Pre-TAVI mean TMG (95% CI)^*^	Post-TAVI mean TMG (95% CI)^*^	Δ mean TMG (95% CI)	*P*-value†
No MAC	1.90 (1.74 to 2.05)	1.97 (1.81 to 2.12)	0.07 (-0.10 to 0.23)	0.92
Mild MAC	2.40 (2.22 to 2.58)	2.61 (2.43 to 2.79)	0.21 (0.01 to 0.40)	0.018
Moderate MAC	3.06 (2.79 to 3.32)	3.36 (3.10 to 3.63)	0.31 (0.02 to 0.60)	0.019
Severe MAC	4.11 (3.80 to 4.42)	4.54 (4.23 to 4.85)	0.43 (0.10 to 0.76)	0.0012

^*^ Estimated marginal means derived from linear mixed models, adjusting for age and sex

† P-values estimated using *t*-tests for pairwise comparisons, adjusted for multiple comparisons using the Tukey method

MAC = mitral annular calcification; TMG = transmitral pressure gradient, mmHg

TTE was performed at a median of 1 [1–38] day before TAVI and 35 [31–45] days after TAVI.

In addition, in a model adjusting for baseline mean TMG, patients with an mean TMG < 5 mm Hg prior to TAVI who had severe MAC were more likely to have an mean TMG > 5 mmHg following the procedure (OR 6.609, 95% CI 1.876 to 23.288, p = 0.003) than those without MAC.

In comparison to the mean TMG, there was no significant interaction between the severity of MAC and time for models evaluating the change in MVA and SVi following TAVI (Table S1, S2). In addition, there was no significant change in heart rate, MVA or SVi following TAVI, irrespective of MAC severity (Table S3, Figures S2, S3).

## Discussion

In this study of more than 700 patients with severe AS who underwent TAVI, the major findings can be summarized as follows: (i) increasing MAC severity (assessed by pre-procedural cardiac CT) was associated with greater increases in mean TMG in the short- to medium- term following TAVI, while there was no significant difference in pre- and post- procedural mean TMG for patients with no MAC; and (ii) there was no significant change in MVA or SVi following TAVI.

### Influence of MAC on mitral valve hemodynamics in patients with severe AS undergoing TAVI

There is now increasing recognition that hemodynamics related to the tubular narrowing of the mitral orifice secondary to MAC (which typically involves the annulus and leaflet bases), vary substantially from those associated with the funnel-shaped mitral orifice of rheumatic heart disease, which is characterized by commissural fusion [17, 18]. Moreover, there is considerable complexity in the evaluation of standard echocardiographic parameters of mitral valve hemodynamics in the presence of MAC. Assessment with planimetry is unreliable due to calcifications, MVA estimations are highly dependent on flow, whilst pressure half-time, mean TMG and MVA are influenced by LV diastolic dysfunction, which is frequently comorbid in patients with MAC [17, 19]. This complexity is further exacerbated in the presence of concomitant severe AS, where there are considerable alterations in LV afterload, LV compliance and LV end-diastolic pressure [20, 21].

MAC is commonly associated with severe AS in patients undergoing TAVI, representing the primary etiology of mitral stenosis in this population [11, 22, 23]. Indeed, in the present study, 58% of patients demonstrated MAC when evaluated by pre-procedural cardiac CT. In light of this considerable prevalence, it is not surprising that an accurate evaluation of MAC may be critical in understanding how mitral valve hemodynamics will be altered following TAVI. In the current analysis, we observed that increasing MAC severity was associated with progressively greater increases in mean TMG following TAVI. Mean TMG is primarily determined by the LA-LV pressure gradient, influenced by atrioventricular compliance, heart rate and stroke volume [14]. As a consequence of the alleviation of LV pressure overload from TAVI, LV compliance is improved and LV end-diastolic pressure reduced, leading to an increase in the LA-LV pressure gradient [24, 25]. However, in patients without MAC, a simultaneous increase in LA compliance following TAVI may reduce LA filling pressures [26], begetting no aggregate change to the LA-LV pressure gradient. In juxtaposition, patients with MAC may not have any appreciable improvement in LA operational compliance following TAVI, due to irreversible atrial fibrotic changes related to increased LA volumes (related to impaired chamber emptying) and associated comorbidities (i.e. hypertension, diabetes and coronary artery disease) [18, 27]. This persistent reduction in LA compliance may prevent any concomitant decrease in LA filling pressures following TAVI, producing an overall increase in the LA-LV pressure gradient and accordingly, the mean TMG. Nonetheless, further research is required to elucidate the precise mechanisms responsible for this finding.

The findings of our study contrast with those of Kato et al [6], who reported a decrease in mean TMG in 58 patients (with a pre-procedural mean TMG ≥ 4 mmHg) following TAVI. However, they did not stratify by MAC and evaluated hemodynamics very early post-procedure (mean 4 [IQR 3–5] days), a period susceptible to perturbations in loading conditions [10]. In a smaller study of 11 patients undergoing TAVI, mean TMG decreased in all patients, with the exception of one patient with severe MAC, in whom the mean TMG increased [5]. This finding was corroborated by the results of the present analysis, where the presence of MAC was associated with an elevated mean TMG following TAVI, at a time interval conducive to the normalization of loading conditions.

Furthermore, we did not observe any significant changes in SVi following TAVI, with results in accordance with the post-hoc analyses of the PARTNER 1 and PARTNER 3 trials [8, 28]. Due to the importance of stroke volume in the continuity equation, this finding may also clarify why no significant change in MVA was observed in the present study, yet previous research had reported a substantial increase in MVA in many patients following TAVI [6]. However, from a pathophysiological perspective, the finding of no change in MVA following TAVI is logical, particularly in patients with MAC, due to the lack of compliance of the mitral annulus and adjacent calcified tissues [18].

### Clinical implications

The current study demonstrates that mean TMG typically increases following TAVI in patients with MAC, suggesting that unlike mitral regurgitation [3, 4], estimates of the hemodynamic severity of degenerative mitral stenosis are likely to worsen, rather than improve, following aortic valve intervention. Indeed, severe AS may conceal the severity of mitral stenosis due to alterations in LV compliance and end-diastolic pressure, secondary to increased afterload. Fortunately, MAC severity is conveniently assessed on standard pre-procedural cardiac CT, providing a method to anticipate changes in degenerative mitral stenosis severity and mean TMG following TAVI. In addition, quantification of the Agatston score of MAC on cardiac CT may offer an alternative method for objective quantification, although further studies are required.

### Limitations

This study is subject to the limitations of its single center, observational and retrospective design. In addition, there was heterogeneity in the timing of follow-up echocardiography (1 to 6 months), although loading conditions have usually normalized across this time interval [10]. Furthermore, the Agatston score of MAC could not be quantified in approximately one-quarter of patients, who did not have a cardiac CT without contrast. In addition, LA fibrosis was not assessed with cardiac magnetic resonance, a parameter that may influence the results of the present study [29].

## Conclusion

In patients with severe AS undergoing TAVI, MAC severity was associated with greater increases in post-procedural mean TMG. In addition, there was no significant difference in pre- and post- procedural mean TMG for patients with no MAC, while there was no significant change in MVA or SVi following TAVI, irrespective of MAC severity.

### Perspectives

#### Competency in medical knowledge

In patients with severe AS, MAC severity assessed with CT may predict the worsening of mean TMG following TAVI. In contrast, no significant alternations of MVA and SVi following TAVI were observed, irrespective of MAC severity. The hemodynamic characteristics of mitral valves with MAC should be taken into consideration when selecting the optimal treatment for patients with severe AS undergoing TAVI.

#### Translational outlook

Increased mean TMG following TAVI in patients with MAC may reflect impaired LA operational compliance and fibrosis. The prognostic implications of mitral valve hemodynamics in patients with MAC undergoing TAVI should be evaluated in future studies.

### Electronic supplementary material

Below is the link to the electronic supplementary material.

Supplementary Material 1

Supplementary Material 2

Supplementary Material 3

Supplementary Material 4

## Abbreviations

AS: Aortic stenosis
CT: Computed tomography
MAC: Mitral annular calcification
MVA: Mitral valve area
SVi: Stroke volume index
TAVI: Transcatether aortic valve initervention
TMG: Transmitral pressure gradient

## References

[1] Okai T, Mizutani K, Hara M, Yamaguchi T, Ogawa M, Ito A, Iwata S, Izumiya Y, Takahashi Y, Shibata T, Yoshiyama M (2020). Presence of mitral stenosis is a risk factor of new development of acute decompensated heart failure early after transcatheter aortic valve implantation. Open Heart.

[2] Nombela-Franco L, Ribeiro HB, Urena M, Allende R, Amat-Santos I, DeLarochelliere R, Dumont E, Doyle D, DeLarochelliere H, Laflamme J, Laflamme L, Garcia E, Macaya C, Jimenez-Quevedo P, Cote M, Bergeron S, Beaudoin J, Pibarot P, Rodes-Cabau J (2014). Significant mitral regurgitation left untreated at the time of aortic valve replacement: a comprehensive review of a frequent entity in the transcatheter aortic valve replacement era. J Am Coll Cardiol.

[3] Toggweiler S, Boone RH, Rodes-Cabau J, Humphries KH, Lee M, Nombela-Franco L, Bagur R, Willson AB, Binder RK, Gurvitch R, Grewal J, Moss R, Munt B, Thompson CR, Freeman M, Ye J, Cheung A, Dumont E, Wood DA, Webb JG (2012). Transcatheter aortic valve replacement: outcomes of patients with moderate or severe mitral regurgitation. J Am Coll Cardiol.

[4] Hutter A, Bleiziffer S, Richter V, Opitz A, Hettich I, Mazzitelli D, Ruge H, Lange R (2013). Transcatheter aortic valve implantation in patients with concomitant mitral and tricuspid regurgitation. Ann Thorac Surg.

[5] Kato N, Shibayama K, Omori N, Hoshina M, Makihara Y, Okumura H, Tabata M, Obunai K, Hirao K, Pellikka PA, Watanabe H (2019). Impact of transcatheter aortic valve replacement on hemodynamic status in patients with aortic stenosis and mitral stenosis: Doppler echocardiographic study. J Cardiol.

[6] Kato N, Padang R, Pislaru C, Miranda WR, Hoshina M, Shibayama K, Watanabe H, Scott CG, Greason KL, Pislaru SV, Nkomo VT, Pellikka PA (2019). Hemodynamics and prognostic impact of concomitant mitral stenosis in patients undergoing Surgical or Transcatheter aortic valve replacement for aortic stenosis. Circulation.

[7] Sannino A, Potluri S, Pollock B, Filardo G, Gopal A, Stoler RC, Szerlip M, Chowdhury A, Mack MJ, Grayburn PA (2019). Impact of mitral stenosis on survival in patients undergoing isolated transcatheter aortic valve implantation. Am J Cardiol.

[8] Hahn RT, Pibarot P, Stewart WJ, Weissman NJ, Gopalakrishnan D, Keane MG, Anwaruddin S, Wang Z, Bilsker M, Lindman BR, Herrmann HC, Kodali SK, Makkar R, Thourani VH, Svensson LG, Akin JJ, Anderson WN, Leon MB, Douglas PS (2013). Comparison of transcatheter and surgical aortic valve replacement in severe aortic stenosis: a longitudinal study of echocardiography parameters in cohort A of the PARTNER trial (placement of aortic transcatheter valves). J Am Coll Cardiol.

[9] Scarsini R, De Maria GL, Joseph J, Fan L, Cahill TJ, Kotronias RA, Burzotta F, Newton JD, Kharbanda R, Prendergast B, Ribichini F, Banning AP (2019). Impact of Complications during Transfemoral Transcatheter aortic valve replacement: how can they be avoided and managed?. J Am Heart Assoc.

[10] Otto CM, Nishimura RA, Bonow RO, Carabello BA, Erwin JP, Gentile F, Jneid H, Krieger EV, Mack M, McLeod C, O’Gara PT, Rigolin VH, Sundt TM, Thompson A, Toly C (2020). 2020 ACC/AHA Guideline for the management of patients with Valvular Heart Disease: a report of the American College of Cardiology/American Heart Association Joint Committee on Clinical Practice Guidelines. Circulation.

[11] Abramowitz Y, Kazuno Y, Chakravarty T, Kawamori H, Maeno Y, Anderson D, Allison Z, Mangat G, Cheng W, Gopal A, Jilaihawi H, Mack MJ, Makkar RR (2017). Concomitant mitral annular calcification and severe aortic stenosis: prevalence, characteristics and outcome following transcatheter aortic valve replacement. Eur Heart J.

[12] Agatston AS, Janowitz WR, Hildner FJ, Zusmer NR, Viamonte M, Detrano R (1990). Quantification of coronary artery calcium using ultrafast computed tomography. J Am Coll Cardiol.

[13] Eberhard M, Schönenberger ALN, Hinzpeter R, Euler A, Sokolska J, Weber L, Kuzo N, Manka R, Kasel AM, Tanner FC, Alkadhi H (2021). Mitral annular calcification in the elderly - quantitative assessment. J Cardiovasc Comput Tomogr.

[14] Baumgartner HC, Hung JC-C, Bermejo J, Chambers JB, Edvardsen T, Goldstein S, Lancellotti P, LeFevre M, Miller F, Otto CM (2017). Recommendations on the echocardiographic assessment of aortic valve stenosis: a focused update from the European Association of Cardiovascular Imaging and the American Society of Echocardiography. Eur Heart J Cardiovasc Imaging.

[15] Lancellotti P, Tribouilloy C, Hagendorff A, Popescu BA, Edvardsen T, Pierard LA, Badano L, Zamorano JL, Scientific Document Committee of the European Association of Cardiovascular I (2013). Recommendations for the echocardiographic assessment of native valvular regurgitation: an executive summary from the European Association of Cardiovascular Imaging. Eur Heart J Cardiovasc Imaging.

[16] Lang RM, Badano LP, Mor-Avi V, Afilalo J, Armstrong A, Ernande L, Flachskampf FA, Foster E, Goldstein SA, Kuznetsova T, Lancellotti P, Muraru D, Picard MH, Rietzschel ER, Rudski L, Spencer KT, Tsang W, Voigt J-U (2015). Recommendations for Cardiac Chamber quantification by Echocardiography in adults: an update from the American Society of Echocardiography and the European Association of Cardiovascular Imaging. J Am Soc Echocardiogr.

[17] Reddy YNV, Murgo JP, Nishimura RA (2019). Complexity of defining severe “Stenosis” from mitral annular calcification. Circulation.

[18] Silbiger JJ (2021). Mitral annular calcification and calcific mitral stenosis: role of Echocardiography in hemodynamic Assessment and Management. J Am Soc Echocardiogr.

[19] Eleid MF, Foley TA, Said SM, Pislaru SV, Rihal CS (2016). Severe mitral annular calcification. JACC: Cardiovasc Imaging.

[20] Villari B, Vassalli G, Monrad ES, Chiariello M, Turina M, Hess OM (1995). Normalization of Diastolic Dysfunction in aortic stenosis late after valve replacement. Circulation.

[21] Dahl JS, Christensen NL, Videbæk L, Poulsen MK, Carter-Storch R, Hey TM, Pellikka PA, Steffensen FH, Møller JE (2014). Left ventricular diastolic function is Associated with Symptom Status in severe aortic valve stenosis. Circ Cardiovasc Imaging.

[22] Mejean S, Bouvier E, Bataille V, Seknadji P, Fourchy D, Tabet JY, Lairez O, Cormier B (2016). Mitral annular calcium and mitral stenosis determined by Multidetector computed tomography in patients referred for aortic stenosis. Am J Cardiol.

[23] Asami M, Windecker S, Praz F, Lanz J, Hunziker L, Rothenbühler M, Räber L, Roost E, Stortecky S, Pilgrim T (2019). Transcatheter aortic valve replacement in patients with concomitant mitral stenosis. Eur Heart J.

[24] Toyota K, Ota T, Nagamine K, Koide Y, Nomura T, Yamanaka F, Shishido K, Tanaka M, Saito S (2016). Effect of transcatheter aortic valve implantation on intraoperative left ventricular end-diastolic pressure. J Anesth.

[25] Gonçalves A, Marcos-Alberca P, Almeria C, Feltes G, Rodríguez E, Hernández-Antolín RA, Garcia E, Maroto L, Fernandez Perez C, Silva Cardoso JC, Macaya C, Zamorano JL (2011). Acute left ventricle diastolic function improvement after transcatheter aortic valve implantation. Eur J Echocardiography.

[26] Poulin F, Thavendiranathan P, Carasso S, Rakowski H, Horlick EM, Osten MD, Cusimano RJ, Woo A (2017). Left atrial phasic function and its Association with Atrial Fibrillation in patients after transcatheter aortic valve implantation. Can J Cardiol.

[27] Morris DA, Gailani M, Vaz Pérez A, Blaschke F, Dietz R, Haverkamp W, Ozcelik C (2011). Left atrial systolic and diastolic dysfunction in heart failure with normal left ventricular ejection fraction. J Am Soc Echocardiography: official publication Am Soc Echocardiography.

[28] Pibarot P, Salaun E, Dahou A, Avenatti E, Guzzetti E, Annabi M-S, Toubal O, Bernier M, Beaudoin J, Ong G, Ternacle J, Krapf L, Thourani VH, Makkar R, Kodali SK, Russo M, Kapadia SR, Malaisrie SC, Cohen DJ, Leipsic J, Blanke P, Williams MR, McCabe JM, Brown DL, Babaliaros V, Goldman S, Szeto WY, Généreux P, Pershad A, Alu MC, Xu K, Rogers E, Webb JG, Smith CR, Mack MJ, Leon MB, Hahn RT (2020). Echocardiographic results of Transcatheter Versus Surgical aortic valve replacement in low-risk patients. Circulation.

[29] Boyle PM, Sarairah S, Kwan KT, Scott GD, Mohamedali F, Anderson CA, Bifulco SF, Ordovas KG, Prutkin J, Robinson M, Sridhar AR, Akoum N (2023). Elevated fibrosis burden as assessed by MRI predicts cryoballoon ablation failure. J Cardiovasc Electrophysiol.

