# Supplementary figures and images for: Hemodynamic implications of mitral annular calcification in patients undergoing transcatheter aortic valve implantation for severe aortic stenosis
Source: Int J Cardiovasc Imaging. 2023 Oct 6;39(11):2183–92. doi: 10.1007/s10554-023-02931-w (PMC10673730; doi:10.1007/s10554-023-02931-w)

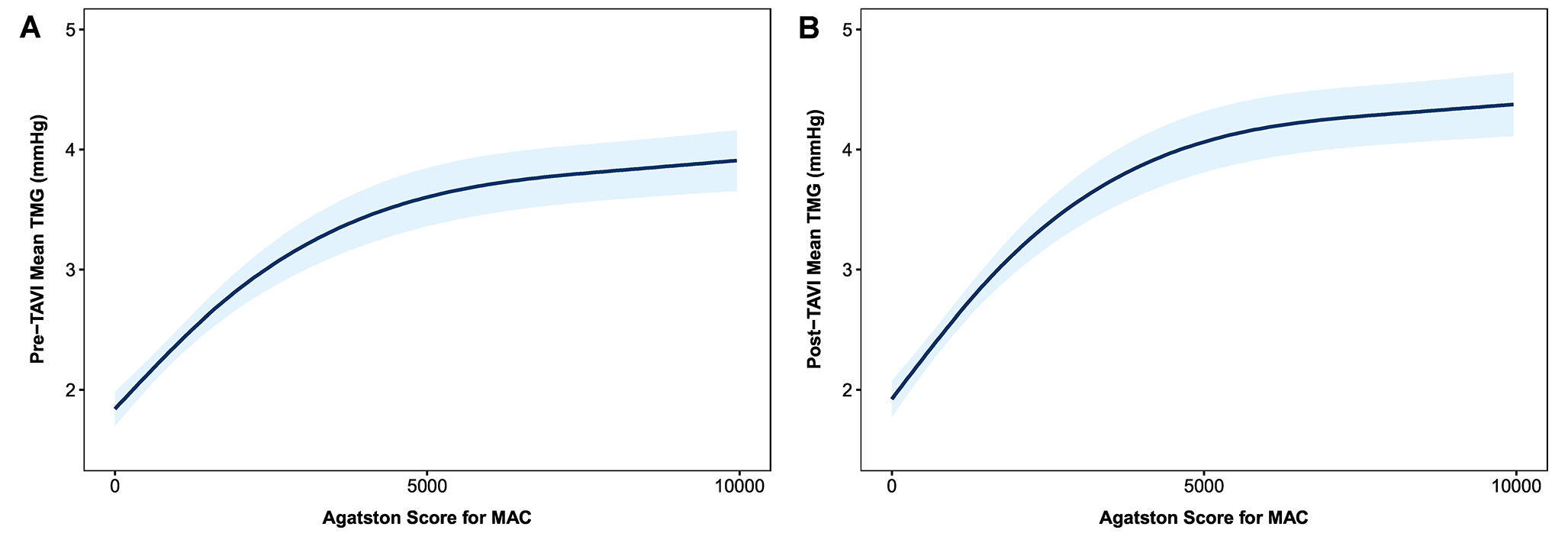

Supplement: Supplementary file 1 — Supplementary Material 1 [file 10554_2023_2931_MOESM1_ESM.png]

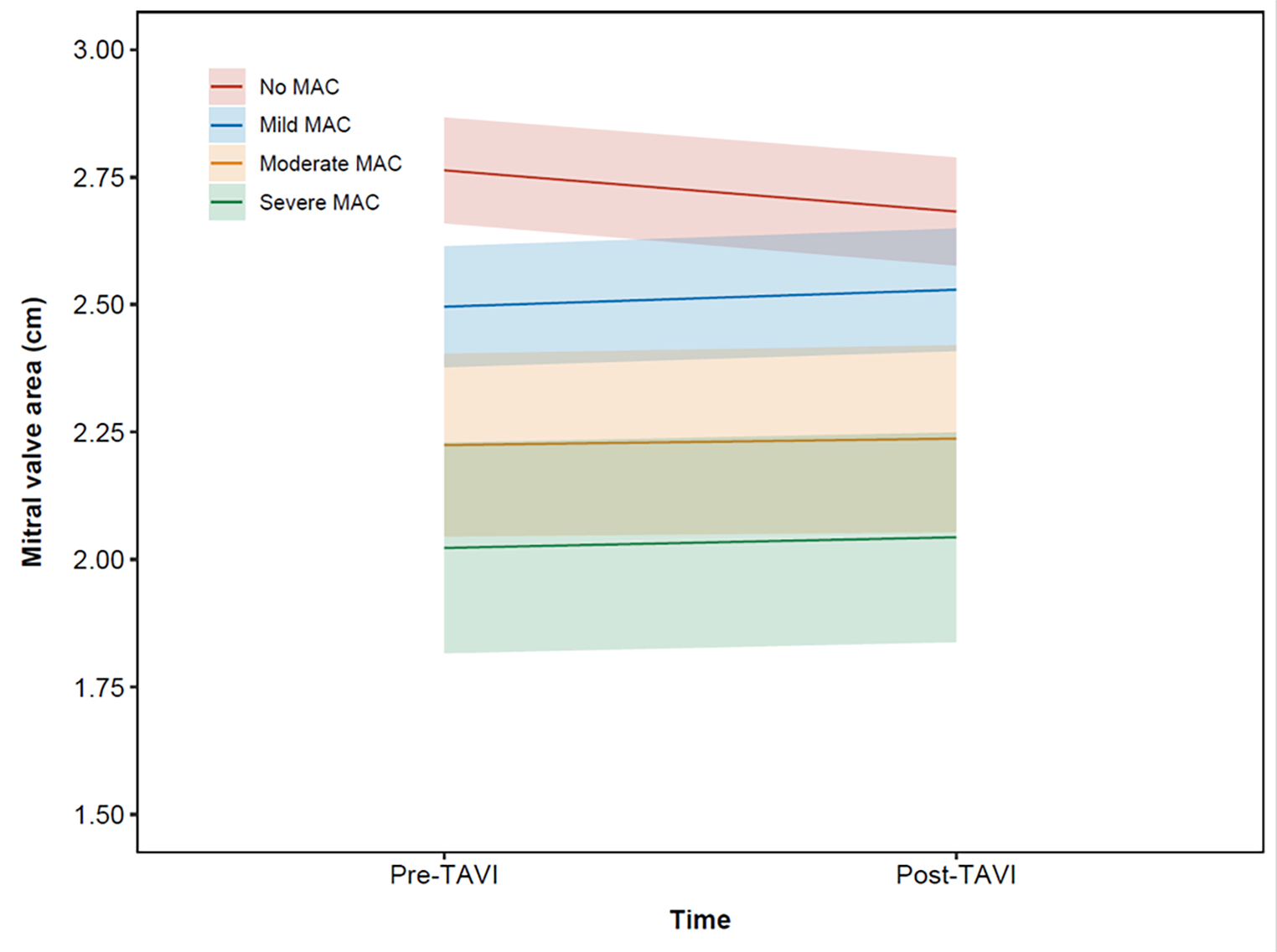

Supplement: Supplementary file 2 — Supplementary Material 2 [file 10554_2023_2931_MOESM2_ESM.png]

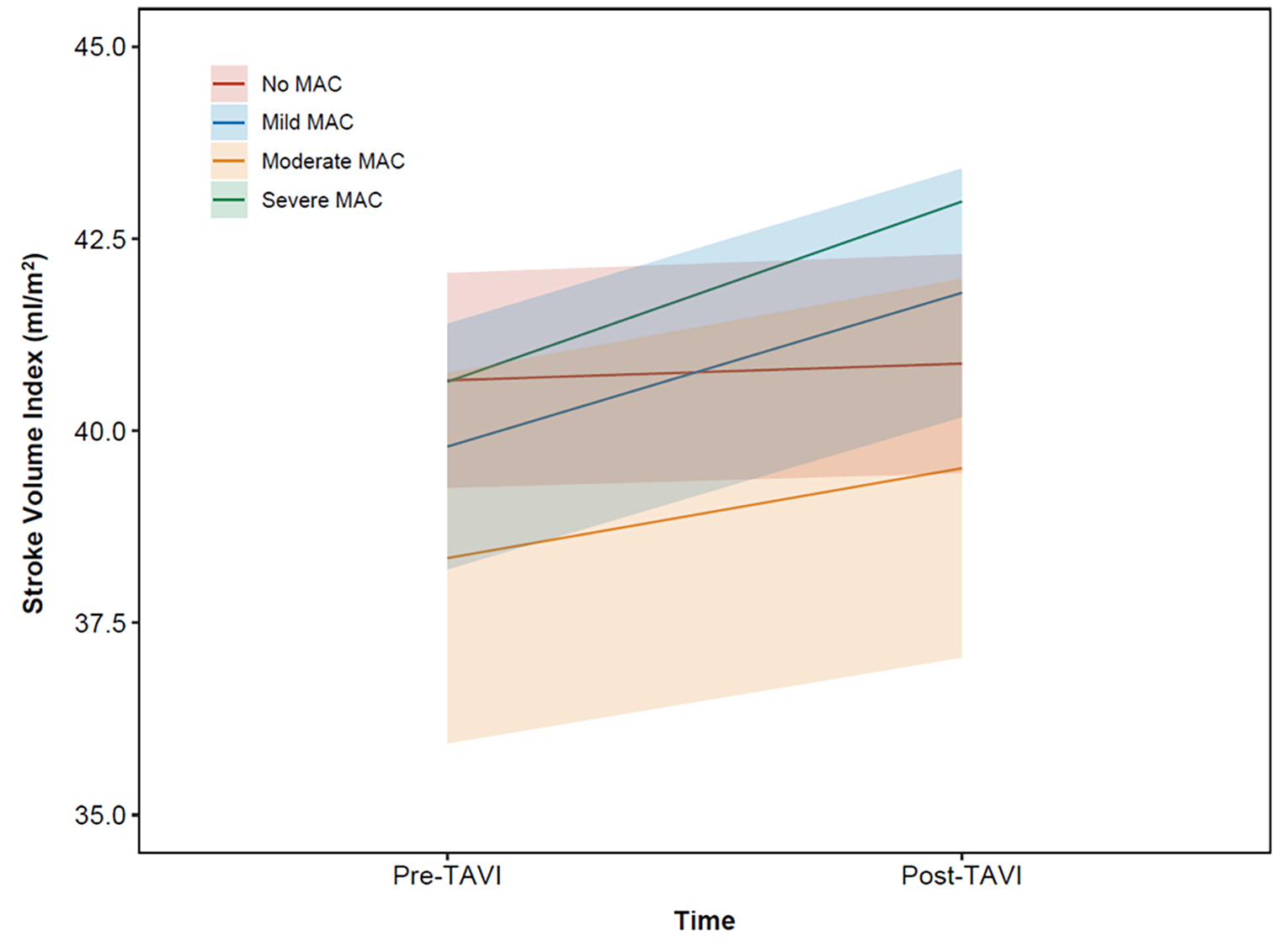

Supplement: Supplementary file 3 — Supplementary Material 3 [file 10554_2023_2931_MOESM3_ESM.png]
