# Supplementary material for: Hemodynamic implications of mitral annular calcification in patients undergoing transcatheter aortic valve implantation for severe aortic stenosis
Source: Int J Cardiovasc Imaging. 2023 Oct 6;39(11):2183–92. doi: 10.1007/s10554-023-02931-w (PMC10673730; doi:10.1007/s10554-023-02931-w)
Supplement: Supplementary file 4 — Supplementary Material 4 [file 10554_2023_2931_MOESM4_ESM.docx]

**Supplemental Figure S1:**

Spline curves demonstrating the association between increasing Agatston score (quantified by Cardiac CT) and values of pre-TAVI (Panel A) and post-TAVI (Panel B) mean TMG, with overlaid 95% confidence intervals (blue-shaded areas).

MAC = mitral annular calcification; TAVI = transcatheter aortic valve implantation; TMG = transmitral gradient.

**Supplementary Tables:**

Table S1: Estimated Marginal Means of MVA according to MAC severity

| **Severity of MAC** | **Pre-TAVI MVA**  **(95% CI)^*^** | **Post-TAVI MVA**  **(95% CI)^*^** | **Δ MVA**  **(95% CI)** | ***P*-value^†^** |
| --- | --- | --- | --- | --- |
| No MAC | 2.76 (2.65 to 2.86) | 2.67 (2.57 to 2.78) | -0.08 (-0.24 to 0.08) | 0.78 |
| Mild MAC | 2.49 (2.37 to 2.61) | 2.52 (2.40 to 2.64) | 0.03 (-0.15 to 0.22) | 1.00 |
| Moderate MAC | 2.22 (2.04 to 2.40) | 2.23 (2.04 to 2.41) | 0.01 (-0.27 to 0.30) | 1.00 |
| Severe MAC | 2.01 (1.81 to 2.22) | 2.04 (1.83 to 2.24) | 0.02 (-0.29 to 0.34) | 1.00 |

^*^ Estimated marginal means derived from linear mixed models, adjusting for age and sex

^†^ *P*-values estimated using *t*-tests for pairwise comparisons, adjusted for multiple comparisons using the Tukey method.

MVA = mitral valve area, cm; MAC = mitral annular calcification.

Table S2: Estimated marginal means of SVi according to MAC severity

| **Severity of MAC** | **Pre-TAVI SVi**  **(95% CI)^*^** | **Post-TAVI SVi**  **(95% CI)^*^** | **Δ SVi**  **(95% CI)** | ***P*-value^†^** |
| --- | --- | --- | --- | --- |
| No MAC | 40.7 (39.3 to 42.1) | 40.9 (39.5 to 42.4) | 0.2 (-1.9 to 2.4) | 1.00 |
| Mild MAC | 39.8 (38.2 to 41.4) | 41.8 (40.2 to 43.5) | 1.14 (-2.3 to 4.6) | 0.17 |
| Moderate MAC | 38.4 (36.0 to 40.8) | 39.6 (37.1 to 42.0) | 1.2 (-2.5 to 4.9) | 0.98 |
| Severe MAC | 40.7 (37.9 to 43.5) | 43.0 (40.3 to 45.8) | 2.3 (-1.8 to 6.5) | 0.63 |

^*^ Estimated marginal means derived from linear mixed models, adjusting for age and sex

^†^ P-values estimated using *t*-tests for pairwise comparisons, adjusted for multiple comparisons using the Tukey method.

MAC = mitral annular calcification; SVi = stroke volume index, ml/m^2^.

**Table S3: Comparison of heart rate at follow-up and change in indexed stroke volume between groups according to MAC severity.**

| Variable | Overall,  N = 708 | No MAC,  N = 299 | Mild MAC,  N = 229 | Moderate MAC,  N = 102 | Severe MAC,  N = 78 | *P*-value |
| --- | --- | --- | --- | --- | --- | --- |
| HR post TAVI (bpm) | 69.1 ± 11.0 | 68.1 ± 10.8 | 69.0 ± 11.3 | 71.2 ± 11.1 | 69.9 ± 10.2 | 0.095 |
| ΔSVi (ml/m^2^) | 1.64 ± 12.1 | 2.95 ± 12.7 | 0.15 ± 11.4 | 1.34 ± 11.4 | 1.43 ± 12.7 | 0.084 |

HR = heart rate; MAC = mitral annulus calcification; SVi = indexed stroke volume; TAVI = transcatheter aortic valve implantation
